# Exploiting Wild Emmer Wheat Diversity to Improve Wheat A and B Genomes in Breeding for Heat Stress Adaptation

**DOI:** 10.3389/fpls.2022.895742

**Published:** 2022-07-22

**Authors:** Mohammed Yousif Balla, Yasir Serag Alnor Gorafi, Nasrein Mohamed Kamal, Modather Galal Abdeldaim Abdalla, Izzat Sidahmed Ali Tahir, Hisashi Tsujimoto

**Affiliations:** ^1^United Graduate School of Agricultural Sciences, Tottori University, Tottori, Japan; ^2^Arid Land Research Center, Tottori University, Tottori, Japan; ^3^Agricultural Research Corporation, Wad Medani, Sudan

**Keywords:** multiple-derivative lines, wild emmer wheat, durum wheat, genome-wide association study, heat

## Abstract

Wheat is highly sensitive to temperature beyond the optimum. To improve wheat adaptation to heat stress, the best option is to exploit the diversity of wild wheat progenitors. This study aimed to identify germplasm and quantitative trait loci associated with heat stress tolerance from wild emmer wheat diversity. We evaluated a diverse set of multiple derivative lines harboring chromosome segments from nine wild emmer wheat parents under four environments: two optimum environments at Tottori, Japan and Dongola, Sudan, one moderate heat stress environment, and one severe heat stress environment at Wad Medani, Sudan. Genome-wide association analysis was conducted with 13,312 SNP markers. Strong marker-trait associations (MTAs) were identified for chlorophyll content at maturity on chromosomes 1A and 5B: these MTAs explained 28.8 and 26.8% of the variation, respectively. A region on chromosome 3A (473.7–638.4 Mbp) contained MTAs controlling grain yield, under optimum and severe heat stress. Under severe heat stress, regions on chromosomes 3A (590.4–713.3 Mbp) controlled grain yield, biomass, days to maturity and thousand kernel weight, and on 3B (744.0–795.2 Mbp) grain yield and biomass. Heat tolerance efficiency (HTE) was controlled by three MTAs, one each on chromosomes 2A, 2B, and 5A under moderate heat stress and one MTA on chromosome 3A under severe heat stress. Some of the MTAs found here were previously reported, but the new ones originated from the wild emmer wheat genomes. The favorable alleles identified from wild emmer wheat were absent or rare in the elite durum wheat germplasm being bred for heat stress tolerance. This study provides potential genetic materials, alleles, MTAs, and quantitative trait loci for enhancing wheat adaptation to heat stress. The derivative lines studied here could be investigated to enhance other stress tolerance such as drought and salinity.

## Introduction

Durum wheat (*Triticum turgidum* L. ssp. *durum*, genome BBAA) is a common tetraploid wheat (2*n* = 28) grown commercially worldwide. Although durum wheat ranks second after bread wheat (*Triticum aestivum* L. ssp. *aestivum*, genome AABBDD) occupying 5% of the total wheat cultivated area (Mastrangelo and Cattivelli, 2021), it has a high market demand because of unique seed characteristics and versatile end uses such as pasta, macaroni, and other semolina products. Moreover, A and B genomes in durum wheat are identical to those of bread wheat, which can be utilized with the D-genome of *Aegilops tauschii* to improve the plasticity of hexaploid wheat, conferring higher adaptation to improve abiotic tolerance and biotic resistance (Rosyara et al., 2019).

Global warming is projected to negatively impact crop growth, development, and productivity in a wide range of environments, posing a threat to global food security (Mishra et al., 2021). Therefore, improving staple food crops to thrive under stressful heat conditions is essential. Wheat is a cold-loving crop that needs daytime temperatures ranging from 17 to 23°C with a nighttime temperature of ≤14°C to give maximum yield potential (John and Megan, 1999; Prasad et al., 2008). Heat above the threshold of 23°C alters various physiological, biological, and biochemical processes in wheat and ultimately decreases grain yield (Akter and Rafiqul Islam, 2017; Djanaguiraman et al., 2020). Grain filling stage is considered the most sensitive stage, where high-temperature stress of 35/20°C (day/night) from 10 days after anthesis until maturity decreased grain yield by 78%, grain number by 63%, and grain weight by 29% (Gibson and Paulsen, 1999). Climate will warm in the coming few decades. An increase of 1°C in temperature is predicted to reduce the average wheat grain yield by 4.1–6.4% (Liu et al., 2016; El Hassouni et al., 2019). Already more than 40% of wheat-growing regions are experiencing increased temperatures above the optimum (Narayanan, 2018; Djanaguiraman et al., 2020). Nevertheless, world wheat production needs to increase by 60% to meet future demand (Zhang et al., 2021). This challenging situation generally requires at least a 1.6% annual wheat yield increases (Narayanan, 2018) and specifically 2.7% increases in semiarid regions where heat waves are prevalent (Iizumi et al., 2021). Therefore, breeding for heat stress tolerance is essential to improve wheat grain yield and adaptation.

Crop breeders usually utilize genetic variation to improve crops against environmental stresses. However, most wheat cultivars have relatively narrow genetic diversity associated with selection on yield *per se*, restricting the potential to breed for heat stress tolerance (Pinto et al., 2017). These constraints imply that the production level of current wheat cultivars cannot fulfill the world demand for food since global population constantly increases. Therefore, a practical solution is to expand the genetic base of wheat by using the adaptive capacity resources of wild progenitors. One of the potential resources is wild emmer wheat (*T. turgidum* ssp. *dicoccoides*), a direct progenitor of domesticated durum wheat (*T. durum*), and the A and B genomes of bread wheat (*T. aestivum*). Wild emmer wheat has been shown to have two lineages that could be exploited for wheat improvement *via* genetic introgression: the western lineage, distributed in Israel, Syria, Lebanon, and Jordan, and the central eastern lineage, distributed in Turkey, Iraq, and Iran (Mori, 2003; Ozkan et al., 2005; Matsuoka, 2011; Peng et al., 2011). Wild emmer wheat is a good resource for improving wheat against environmental stresses (Peng et al., 2013; Tsujimoto et al., 2015; El Haddad et al., 2020). Interestingly, the natural variation of wild emmer wheat encompasses important agronomic-, physiological-, and yield-related traits associated with heat stress tolerance (Peng et al., 2013). Thus, this diversity in wild emmer wheat is needed to sustain and improve wheat tolerance against heat stress.

Heat stress tolerance is a complex trait strongly affected by the environment, and genotype-by-environment interaction seriously restricts its selection for sustainable breeding. Hence, breeding to improve heat stress tolerance should be conducted in multiple environments to provide evidence of the stability of the key traits (Liu et al., 2019a; Ma’arup et al., 2020).

Most of the previous research targeted exploiting the diversity in wild wheat progenitors to improve heat stress tolerance in hexaploid wheat (Elbashir et al., 2017; Liu et al., 2019b; Ma’arup et al., 2020; Elhadi et al., 2021a,b; Itam et al., 2021a,b; Ullah et al., 2021). However, in durum wheat, only a pre-breeding set of 77 advanced lines has been developed and evaluated for heat stress tolerance (Aberkane et al., 2020, 2021). Also, a field evaluation for heat stress tolerance has been conducted on a diverse set of elite lines and landraces (Sall et al., 2018; El Haddad et al., 2020). On the other hand, genetic information from tetraploid wheat growing under field conditions is rare (Sukumaran et al., 2018; El Hassouni et al., 2019; Wang et al., 2019). Although previous work identified many quantitative trait loci (QTLs) associated with heat stress tolerance in tetraploid wheat evaluated under various climatic conditions (Sukumaran et al., 2018; El Hassouni et al., 2019), such QTLs associated with wild emmer wheat diversity have not been fully explored in a modern durum wheat background. Our previous research work described the creation and development of multiple derivative lines (MDLs) by crossing and backcrossing nine wild emmer wheat accessions with the durum wheat cultivar “Miki 3” (Balla et al., 2022). This population harbors in its gene pool genomic fragments from both wild emmer wheat lineages. With the expectation that the diversity in the MDLs could be used to improve heat stress tolerance in tetraploid wheat, in the current study, we evaluated a diverse set of 178 MDLs under four environments, including optimum and heat stress conditions. The objective was to identify QTLs linked to heat stress tolerance from the wild emmer wheat diversity in the MDLs, and germplasm to be used in breeding for heat stress tolerance. The MDL platform used in this study provides valuable genetic materials and QTLs to improve both bread wheat and durum wheat adaptation to heat stress conditions.

## Materials and Methods

### Plant Material

We used a population of multiple derivative lines (MDLs) consisting of 178 BC_1_F_6_ durum wheat (*Triticum turgidum* ssp. *durum*) lines, and their recurrent parent “Miki 3.” The 178 BC_1_F_6_ lines were developed by crossing and backcrossing nine wild emmer wheat (*T. turgidum* ssp. *dicoccoides*) accessions with the common durum wheat cultivar “Miki 3.” Detailed information for MDL development and population structure are available in Balla et al. (2022).

### Field Evaluation and Experimental Design

Field experiments were conducted during the winter season (2019–2020) at four sites: one in Japan and three in Sudan. All experiments were arranged in an alpha lattice design with two replications. In Japan, the experiment was conducted in Tottori at the field of the Arid Land Research Center, Tottori University (35°32′N, 134°13′E, 11 m a.s.l.), hereinafter abbreviated TOT. Seeds were planted and germinated in tray pots in November and transferred to the field during the first week of December and harvested in mid-June. Each genotype was planted in one row of five plants with 0.2 m spacing between plants and 0.8 m spacing between rows. This location has a cold winter with rain-fed field conditions, and the total rainfall amount during the experiment was 930 mm (Arid Land Research Center weather station). All field descriptions and management were the same as described in Elhadi et al. (2021a).

In Sudan, the first experiment was conducted at Dongola Research Station Farm, in North Sudan (19°08′N, 30°27′E, 239 m a.s.l; abbreviated DON). The second and third experiments were conducted at the Gezira Research Station Farm (GRSF), Agricultural Research Corporation, Wad Medani (14°24′N, 29°33′E, 407 m a.s.l.) with optimum (MED/SD1) and late sowing (MED/SD2), respectively. The late sowing was performed to ensure exposure of the plants to heat stress during the reproductive stage. The GRSF is located in the central clay plain in Gezira State.

In all sites, seeds of each genotype were sown manually in a plot consisting of four rows of 1.0 m length with 0.2 m spacing between rows (the total number of plants per row was about 60 plants). In Dongola, the sowing was during the first week of December, while in Wad Medani, the optimum or first sowing date was in the 4th week of November and the late or second sowing date was in the 4th week of December. All field descriptions, management, seed treatments, and fertilization were the same as described in Itam et al. (2021b). In Sudan, there is no rainfall during the winter season, and irrigation was carried out at 10–14 day intervals (wheat requires about 400 mm water) to avoid water stress. All cultural practices followed the Agricultural Research Corporation’s recommendations for wheat production in Sudan.

### Measurement of Phenological, Leaf Physiological, and Grain Yield Traits

Phenological traits included days to heading (DH) observed as the number of days from first irrigation or transplanting until 50% of the plant reached heading. Days to maturity (DM) was recorded when 90% of plants lost green color from the glumes. Grain filling duration (GFD) was calculated as the difference in days between DH and DM. Plant height (PHT, cm) was recorded at maturity by measuring the distance between the ground and the top of the spike, excluding awns.

Leaf physiological traits included chlorophyll content at heading (CHLH) and chlorophyll content at maturity (CHLM) measured from three random flag leaves per plot using the Minolta SPAD-502 chlorophyll meter (Konica Minolta, Japan). Chlorophyll content degradation (CHLD) was calculated as the ratio between CHLM and CHLH (100*CHLM/CHLH). Canopy temperature at heading (CTH) was measured from the canopy of each plot from 13:00 to 14:00 using a handheld infrared thermometer (Everest Interscience, Tucson, AZ, United States) only in Sudanese environments.

Yield and its component traits included grain yield (GY), biomass (BIO), thousand kernel weight (TKW), harvest index (HI), and seed number/spike (SN). GY was determined as grain weight per plot and then converted to kg ha^−1^ for further analysis. BIO was measured for the above-ground dry weight per plot and converted to kg ha^−1^ for further analysis. TKW (g) and SN were determined from random samples of 10 spikes taken from the central rows. HI was measured as the ratio between BIO and GY (GY/BIO*100).

### Statistical Analysis of Phenotypic Data

Analysis of variance (ANOVA) for alpha lattice design of all studied traits in each location was performed in GenStat 18th edition.^1^ We used Tukey’s honestly significant difference (HSD) test for environment–environment comparisons. Pearson’s correlation coefficient between traits in each environment was calculated in IBM SPSS Statistics for Windows v. 25 (IBM Corp., Armonk, NY, United States). Broad-sense heritability was estimated in Plant Breeding Tools v. 1.4.^2^

To identify heat stress-tolerant genotypes, heat tolerance efficiency (HTE) was calculated as 100*(Y_si_/Y_pi_), where Y_si_ is GY under stress or in a hot environment and Y_pi_ is GY under an optimum or cold environment (Elbashir et al., 2017). In the first HTE (HTE1), we used the GY values from DON as the cold environment and MED/SD1 as the hot environment. In the second HTE (HTE2), we considered GY values from MED/SD1 as the cold environment and MED/SD2 as the hot environment.

### SNP Genotyping and Data Analysis

Total genomic DNA of all genotypes was extracted following a modified CTAB method (Saghai-Maroof et al., 1984). DNA samples (20 μl; 50–100 ng μL^−1^) were sent to Diversity Array Technology (DArT) Pty., Ltd., Australia^3^ for whole-genome scanning with the DArTseq (DArT sequencing) platform. Restriction fragments from each sample were sequenced and aligned to durum wheat cv. “Svevo” RefSeq v.1.0 to generate SilicoDArT or SNP markers (Maccaferri et al., 2019). We obtained 32,942 mapped SNP markers scored as “0” (homozygous reference allele), “1” (homozygous SNP allele), or “2” (heterozygous) with a call rate of 90% (10% missing genotype). After removing markers with minor allele frequency of <0.05, we obtained 13,312 SNPs markers and used them for genome-wide association (GWA) analysis.

### GWA Analysis and Candidate Genes Identification

GWA analysis was performed for each location separately using a mixed linear model (MLM) implemented in TASSEL v. 5.6 (Bradbury et al., 2007). The MLM approach was used to control population structure and relatedness effects and reduce the rate of false-positive associations. The significant marker-trait associations (MTAs) were detected at the threshold −log_10_ (*P*) > 3. The values of *p* were adjusted for multiple testing using two-levels false discovery rate (FDR) at 0.05 and 0.2 (Benjamini and Hochberg, 1995). The MLM product from TASSEL was used in R v. 4.0.3 with custom scripts in the developed GWAS package rMVP to draw Manhattan plots and quantile−quantile plots for GY (Yin et al., 2021). MTAs were considered stable when found in two or more environments and considered pleiotropic when detected for two or more traits. The favorable alleles for each QTL region were identified by comparing the significant MTAs with extreme phenotypic values in the MDL panel.

For candidate genes analysis, we selected the highly significant markers of the important traits evaluated under the optimum condition at DON, moderate heat (MED/SD1), and severe heat (MED/SD2) stress. We searched for candidate genes in a ± 0.5 Mbp window size from the position of the significant marker by blasting the sequence of the significant markers against IWGSC RefSeq v 2.1 database.^4^ We used Ensemble Plant database^5^ to determine the number and name of the known genes. The functions of the putative genes were identified using UniProtKB.^6^

## Results

### Climate Condition

**Figure 1:**
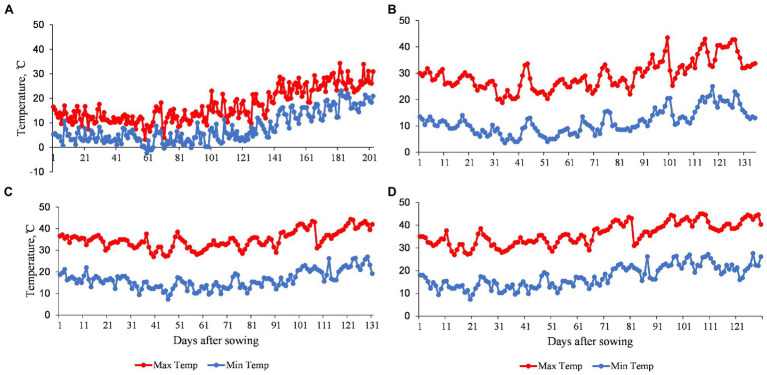
Daily maximum and minimum air temperatures of the four environments used to evaluate the multiple derivative lines (MDLs): **(A)** Tottori (TOT); **(B)** Dongola (DON); **(C)** Wad Medani first sowing date (MED/SD1); and **(D)** Wad Medani second sowing date (MED/SD2).

Temperature data of the four environments used to evaluate the MDLs during the 2019–2020 growing season are shown in Figure 1. At TOT, the average daily maximum and minimum air temperatures were 17.0°C and 7.8°C, respectively (Figure 1A). At DON, the average daily maximum and minimum air temperatures were 29.3°C and 11.4°C, respectively (Figure 1B). However, at MED/SD1, the average daily maximum and minimum temperatures were 34.9°C and 16.3°C (Figure 1C), while at MED/SD2, they were 36.2°C and 17.4°C, respectively. MED/SD2 experienced more heat stress mainly at the reproductive stage (Figure 1D). In TOT, there was no heat stress during the growing season. DON was the coolest among the Sudanese environments and MED/SD1 was cooler than MED/SD2. Therefore, we considered TOT and DON as favorable environments, and MED/SD1 and MED/SD2 as moderate and severe heat stress environments, respectively.

### Effect of Heat Stress on MDL Performance

**Figure 2:**
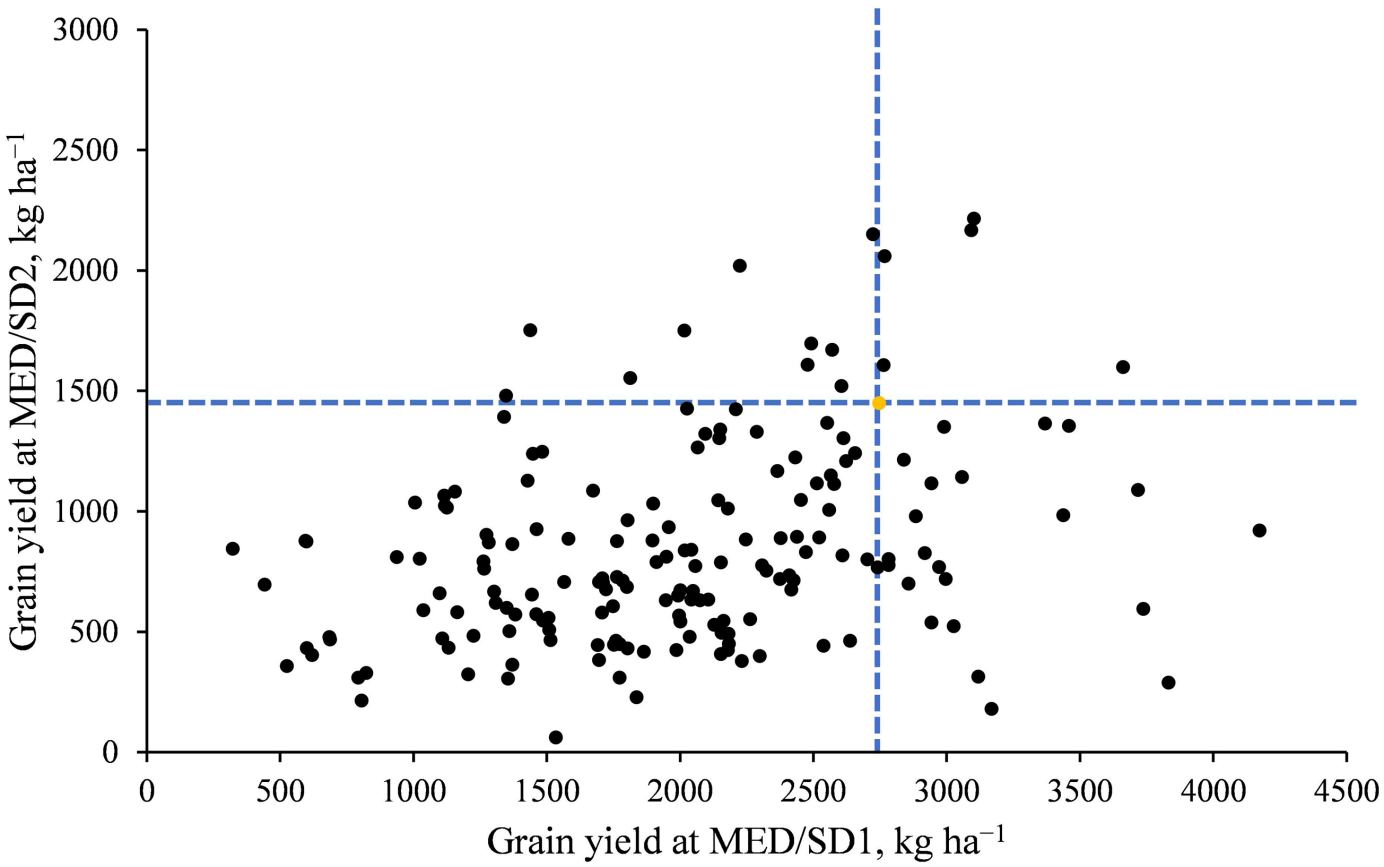
Average grain yield of the MDLs in Wad Medani for the second sowing date (MED/SD2) versus that in Wad Medani for the first sowing date (MED/SD1). The dashed blue lines intersect on the recurrent parent “Miki 3” (yellow circle). A few MDLs showed higher grain yield than their recurrent parent ‘Miki 3’ for both MED/SD1 and MED/SD2.

The mean GY was 4,838 kg ha^−1^ in TOT, 3691 kg ha^−1^ in DON, 2018 kg ha^−1^ in MED/SD1, and 848 kg ha^−1^ in MED/SD2, indicating the severity of heat stress in Wad Medani (Supplementary Figure S1; Table S1). Heat stress (from TOT or DON to MED/SD1 or MED/SD2) caused significant (*p* < 0.001) decreases in GY, BIO, TKW, SN, HI, PHT, DH, DM, GFD, CHLH, and CHLM, and significant (*p* < 0.001) increases in CTH (Supplementary Figure S1). Late sowing at Wad Medani significantly (*p* < 0.001) reduced CHLD and increased CHLM compared with DON (Supplementary Figure S1). The ANOVA showed a highly significant (*p* < 0.001) genotype (G) effect for most of the studied traits in all environments (Table 1). The genotype-by-environment (G × E) interaction effects were highly significant (*p* < 0.0001) for all studied traits except CTH and HI. The broad-sense heritability estimates were high (0.91 to 0.68) for DH, DM, TKW, GFD, and PHT, indicating significant genetic control for these traits (Table 1). However, GY, BIO, SN, and HI showed moderate heritability estimates ranging from 0.38 for GY to 0.48 for SN. In contrast, the lowest heritability estimate of 0.10 was observed for CHLM. Despite the heat stress severity in Wad Medani, some MDLs showed higher GY than their recurrent parent “Miki 3” in MED/SD1 and MED/SD2 (Figure 2).

**Table 1:** Sum of squares of the 13 evaluated traits in the multiple derivative lines grown at Tottori (TOT), Dongola (DON), Wad Medani first sowing date (MED/SD1) and Wad Medani second sowing date (MED/SD2).

Trait	TOT	DON	MED/SD1	MED/SD2	Combined			
	G	G	G	G	G	E	G × E	*h^2^*
DH	48.6^***^	32.0^***^	62.9^***^	32.5^***^	148.0^***^	402276.2^***^	18.9^***^	0.91
DM	8.6^***^	29.5^***^	42.1^***^	27.5^***^	90.8^***^	497786.7^***^	15.9^***^	0.85
GFD	43.5^***^	22.8^***^	17.4^***^	8.1^**^	39.5^***^	10456.7^***^	17.9^***^	0.70
CHLH	28.74^ns^	97.9^**^	13.7^***^	8.7^*^	47.9.0^***^	7290.0^***^	35.3^*^	0.51
CHLM	67.75^**^	208.7^***^	9.6^*^	5.1^ns^	78.6^***^	67064.5^***^	76.2^***^	0.10
CHLD	273.2^ns^	750.7^**^	64.5^ns^	15.1^ns^	283.3^*^	226344.3^***^	294.7^***^	
CTH		7.6^ns^	9.2^*^	5.2^*^	12.4^ns^	4622.7^***^	13.4^ns^	0.49
GY	3626957.0^***^	1482059.0^**^	983349.0^***^	321533.0^***^	2058936.0^***^	1114870046.0^***^	1650547.0^***^	0.38
BIO	23033923.0^***^	21186611.0^*^	8935496.0^***^	7139663.0^***^	21380000.0^***^	9861000000.0^***^	15890000.0^***^	0.43
TKW	41.0^***^	74.1^**^	47.65^**^	52.2^**^	83.4^***^	13565.2^***^	43.3^***^	0.72
SN	136.6^***^	74.5^***^	91.9^***^	92.0^***^	128.8^***^	19273.8^***^	94.0^***^	0.48
HI	42.35^***^	233.9^ns^	103.8^***^	56.0^***^	133.5^***^	15123.1^***^	102.5^ns^	0.47
PHT	132.5^***^	282.9^***^	114.6^*^	89.5^ns^	256.5^***^	64867.7^***^	128.9^***^	0.68

^*^, ^**^, ^***^, and ^ns^ indicate F-probability values less than 0.05, 0.01, 0.001 and non-significant, respectively. DH, days to heading; DM, days to maturity; GFD, grain filling duration; CHLH, chlorophyll at heading; CHLM, chlorophyll at maturity; CHLD, chlorophyll degradation; CTH, canopy temperature at heading; GY, grain yield; BIO, biomass; TKW, thousand kernel weight; SN, seed number/spike; HI, harvest index; PHT, plant height. G, indicates the main genotype effect at each environment or combined environment; E, environment main effect; G × E, genotype by environment interaction; h^2^ heritability estimate.

**Figure 3:**
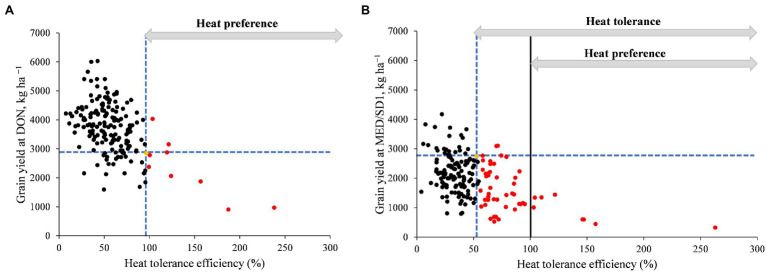
Grain yield (GY) versus heat tolerance efficiency (HTE). **(A)** GY at Dongola (DON) versus HTE calculated using the GY of DON as the cool environment and that of Wad Medani for the first sowing date (MED/SD1) as the hot environment (referred to as HTE1 in the text). **(B)** GY at MED/SD1 and HTE calculated using the GY of MED/SD1 as the cool environment and that of Wad Medani for the second sowing date MED/SD2 as the hot environment (referred to as HTE2 in the text). The yellow dot indicates the values for ‘Miki 3’. Black dots denote MDLs with HTE less than that of ‘Miki 3’. Red dots denote MDLs with HTE higher than that of ‘Miki 3’. Dashed blue lines intersect on ‘Miki 3’. Vertical black line marks HTE = 100. Horizontal grey arrows denote the ranges of heat tolerance and heat preference.

We calculated HTE to identify heat-tolerant genotypes. As grain yield is important for breeding, we performed regression analysis between GY and HTE to identify the heat-tolerant genotypes with respect to their yield potential (Figure 3). In the first HTE (HTE1, calculated from GY of DON and MED/SD1), nine genotypes (5%) had a higher HTE1 than their recurrent parent “Miki 3” (Figure 3A). However, in the second HTE (HTE2, calculated from GY of MED/SD1 and MED/SD2), 25% of the MDLs had a higher HTE2 than their recurrent parent “Miki 3,” indicating high genetic gain (Figure 3B). Most of the MDLs that showed higher HTE in both HTE1 and HTE2 had a lower GY than “Miki 3” except for four genotypes (Figures 3A,B).

GY correlated significantly (*p* < 0.05) with most of the studied traits in the four environments (Supplementary Tables S2−S5). In all environments, GY showed a consistent correlation (*p* < 0.01) with BIO and SN, with correlation (*r*) values ranging from 0.225 to 0.836. GY was positively correlated with HI in all environments except DON. In contrast, no leaf physiological traits were correlated with GY except CTH in MED/SD2, which revealed a significant negative correlation of −0.220 (Supplementary Table S5). DH and GFD were negatively correlated in all environments, with correlation (*r*) values ranging from −0.382 to −0.899 (Supplementary Tables S2−S5). Under both heat stress conditions, MED/SD1 and MED/SD2, HTE values were positively correlated with GY, BIO, HI, as well as with CHLM in MED/SD2 (Supplementary Tables S4, S5).

### Detection of MTAs

**Figure 4:**
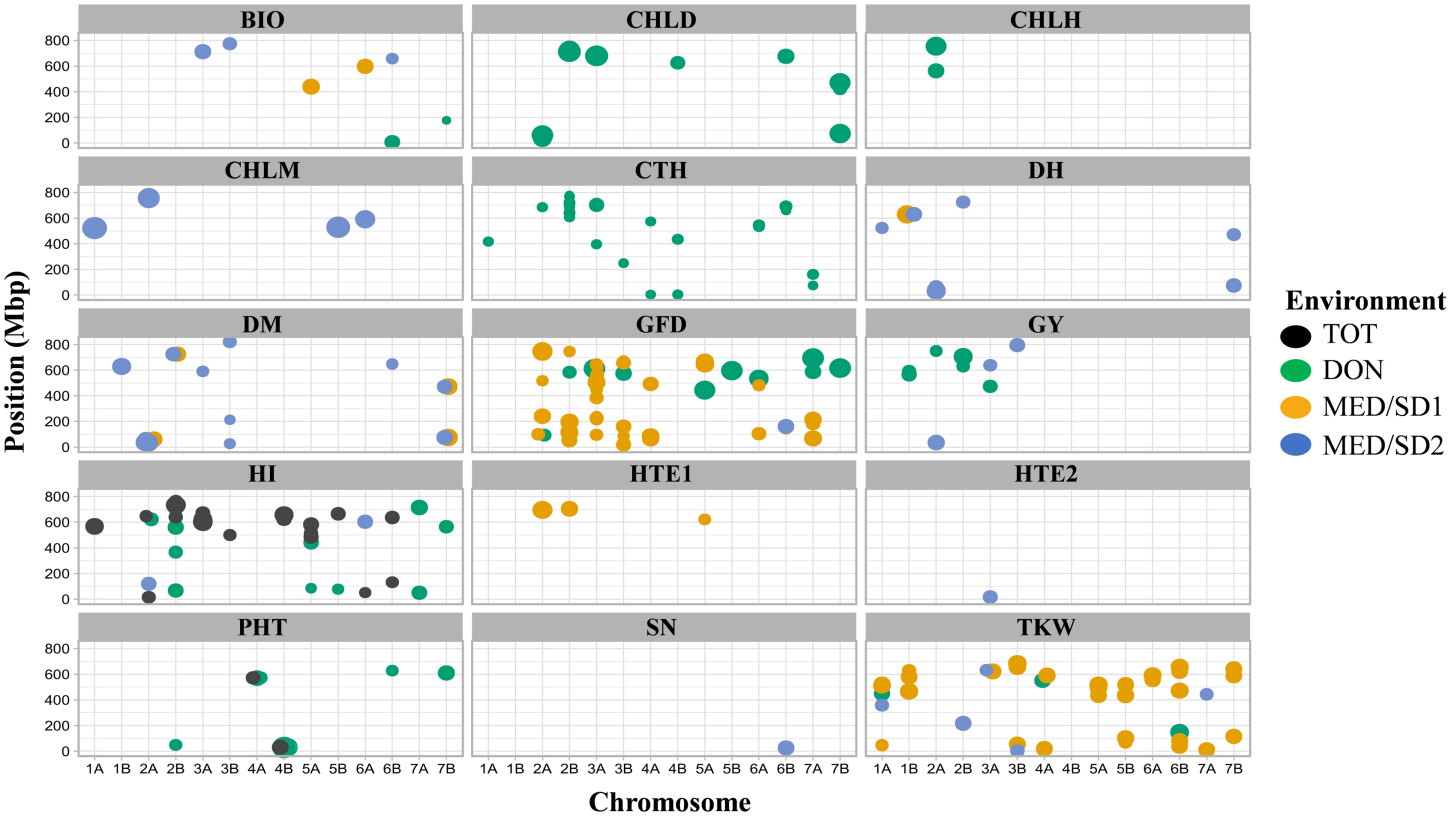
Physical positions of markers associated with evaluated traits in the four environments; Tottori (TOT); Dongola (DON); Wad Medani first sowing date (MED/SD1); and Wad Medani second sowing date (MED/SD1). BIO, biomass; CHLD, chlorophyll degradation; CHLH, chlorophyll at heading; CHLM, chlorophyll at maturity; CTH, canopy temperature at heading; DH, days to heading; DM, days to maturity; GFD, grain filling duration; GY, grain yield; HI, harvest index; HTE1, heat tolerance efficiency evaluated in MED/SD1; HTE2, heat tolerance efficiency evaluated in MED/SD2; PHT, plant height; SN, seed number/spike; TKW, thousand kernel weight. Symbol size corresponds to the allelic effect of each MTA.

At −log_10_ (*P*) > 3, a total of 287, 427, 299, and 406 MTAs were detected in TOT, DON, MED/SD1, and MED/SD2, respectively (Supplementary Tables S6−S9). To reduce false positives, we used a more stringent FDR threshold of 0.05 and 215 MTAs were identified across environments (Supplementary Table S10). However, because this FDR threshold is stringent and many potentially important MTAs were excluded, we reduced the threshold up to 0.2, and identified an additional 90 MTAs (Supplementary Table S10). In total, 35 highly significant MTAs were identified in TOT (30 MTAs with FDR of 0.05 and 5 with FDR of 0.2), 117 in DON (64 MTAs with FDR of 0.05 and 53 with FDR of 0.2), 102 in MED/SD1 (101 MTAs with FDR of 0.05 and one MTA with FDR of 0.2), and 51 in MED/SD2 (21 MTAs with FDR of 0.05 and 30 with FDR of 0.2; Figure 4; Supplementary Table S10). The MTAs for each trait varied, with the highest number observed for GFD (59), followed by TKW (45), HI (44), and PHT (30; Supplementary Figure S2A). In all environments, the number of MTAs observed on the A genome (156) and B genome (149) was almost similar (Supplementary Figure S2 B). The highest number of MTAs was observed on chromosomes 2A (51) and 2B (41), and the lowest number on chromosome 1B (9; Supplementary Figure S2 B). To avoid confounding effects of phenological genes on different traits, GWA analysis was performed for GY and other traits for each location using DH as a covariate (Sukumaran et al., 2018). All the identified MTAs were independent of the effect of phenological genes except HTE2.

### MTAs Under Favorable Conditions (TOT and DON)

In TOT, significant MTAs were detected for HI and PHT (Figure 4; Supplementary Table S10), whereas no significant associations were detected for the other traits at the FDR thresholds. The 32 MTAs identified for HI were scattered on 10 chromosomes, and 20 of them were collocated. Five MTAs collocated on chromosomes 2B (635–765 Mbp), 3A (598–672 Mbp), 4B (626–658 Mbp), and 5B (664–671 Mbp) explained on average 13.7, 16.3, 13.9, and 12.0% of the phenotypic variation, respectively. Three MTAs were detected for PHT on chromosomes 4A (one MTA at 572.5 Mbp) and 4B (2 MTAs at 30.5–31.3 Mbp), which explained 10.9–15.2% of the variation (Figure 4; Supplementary Table S10).

In DON, 57.0% of the MTAs detected were for CTH, PHT, CHLD, GFD, and HI (Supplementary Table S10). The 29 MTAs detected for PHT were distributed on chromosomes 2B, 4A, 4B, 6B, and 7B; these MTAs explained from 11.1 to 26.6% of the phenotypic variation. Of these MTAs, 21 were collocated on chromosome 4B (12.2–57.5 Mbp; Figure 4; Supplementary Table S10). Among 36 MTAs for CTH, 13 were collocated on chromosome 2B (608.7–781.3 Mbp) and explained 8.6–10.5% of the phenotypic variation (Figure 4; Supplementary Table S10).

**Figure 5:**
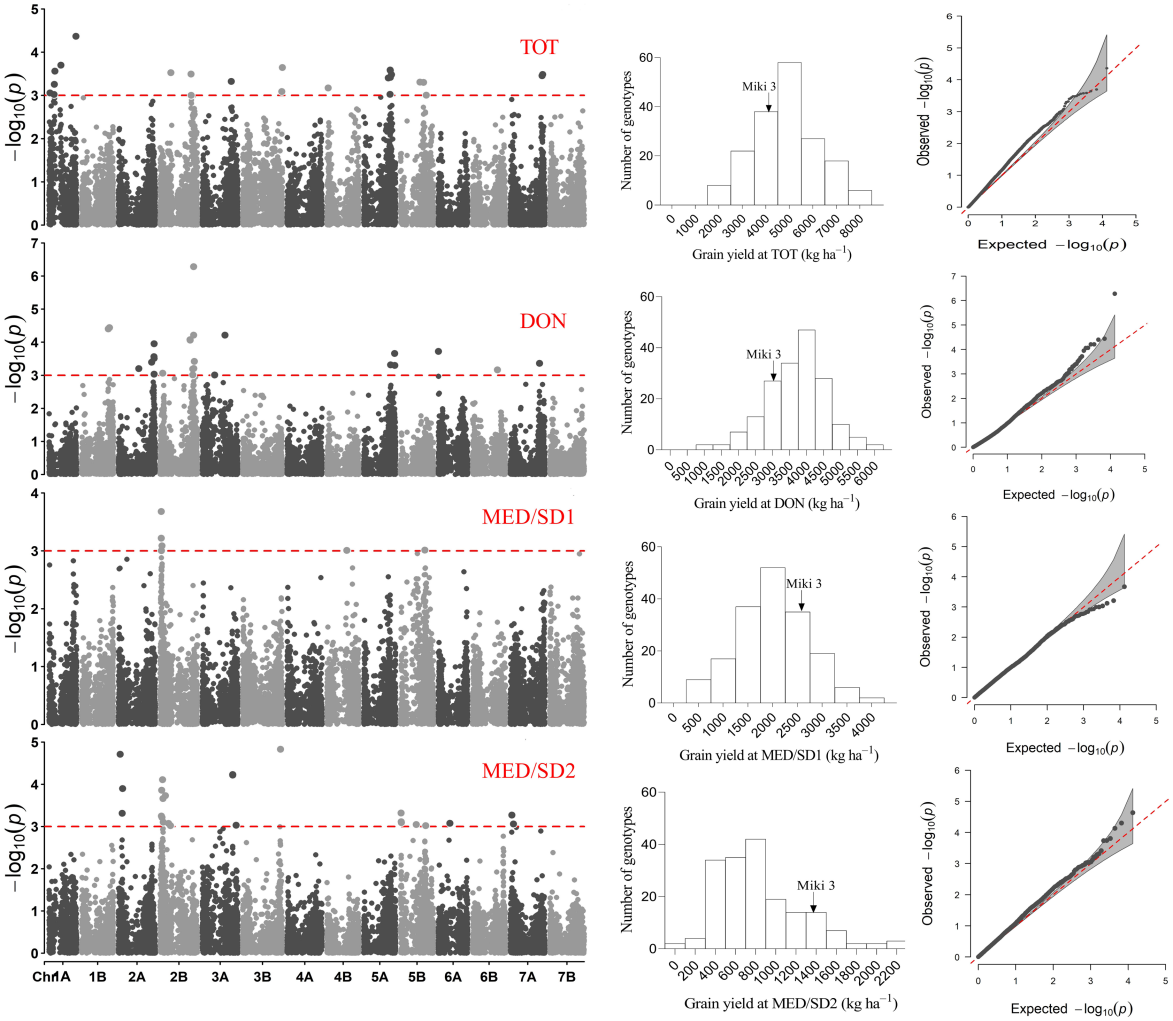
Representative Manhattan plots for grain yield of the genome-wide analysis showing marker-trait association in the four environments; Tottori (TOT), Dongola (DON), Wad Medani first sowing date (MED/SD1) and Wad Medani second sowing date (MED/SD2). Frequency distributions and quantile−quantile plots for grain yield are shown for each environment.

**Figure 6:**
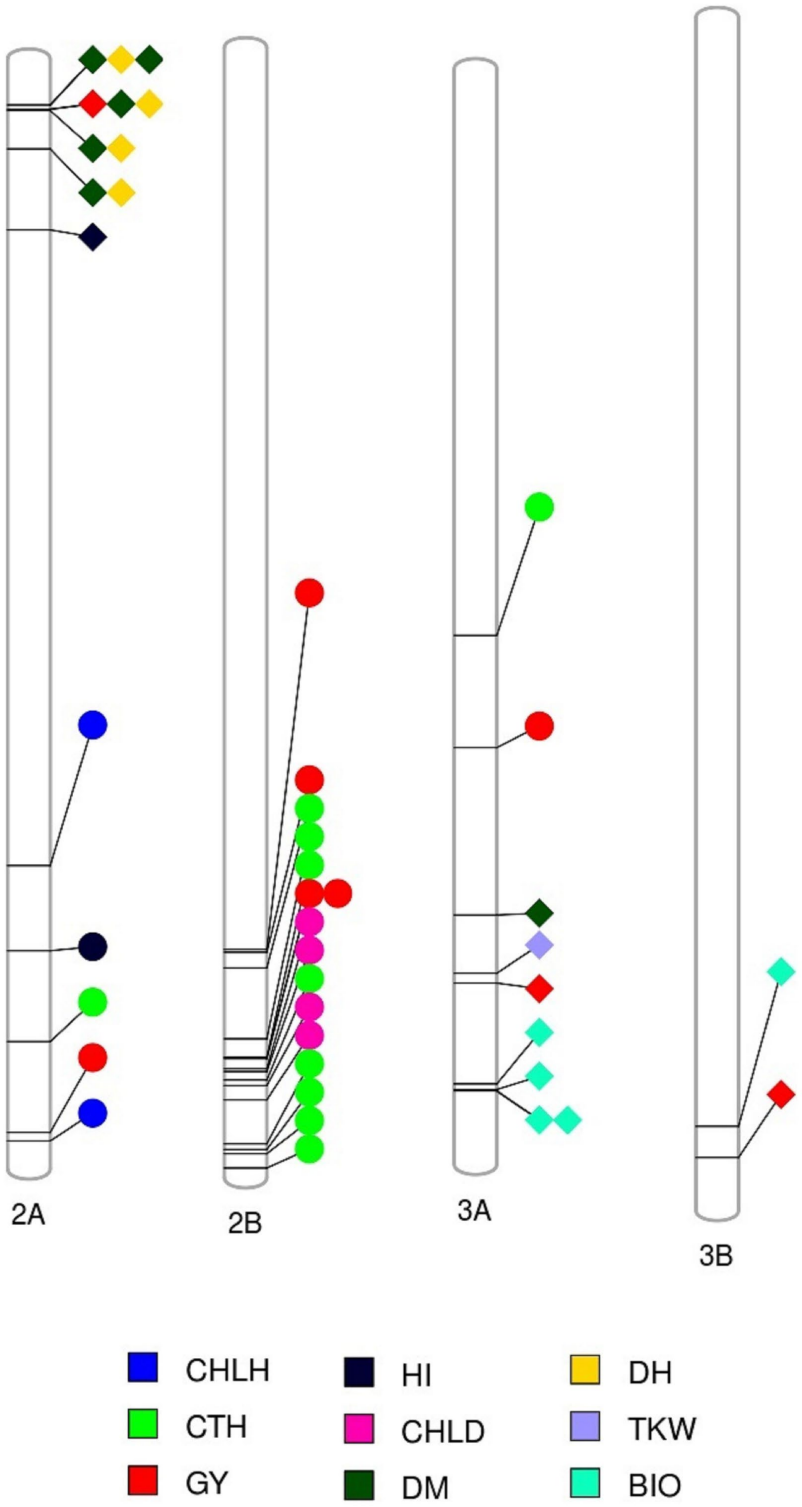
Markers associated with multiple traits (see color key legend in the figure) identified under favored environment at Dongola (circles) or severe heat stress environment at Wad Medani (MED/SD2; diamonds). BIO, biomass; CHLD, chlorophyll degradation; CHLH, chlorophyll at heading; CTH, canopy temperature at heading; DH, days to heading; DM, days to maturity; GY, grain yield; HI, harvest index; TKW, thousand kernel weight.

The eight MTAs for GY were located on chromosomes 1B (2 MTAs at 565.8–590.4 Mbp), 2A (1 MTA at 749.6 Mbp), 2B (4 MTAs at 629.4–705.2 Mbp), and 3A (1 MTA at 473.7 Mbp) that explained from 11.2 to 18.3% of the phenotypic variation (Figures 4, 5). A region on chromosome 2A (563.6–755.9 Mbp) had significant MTAs for CHLH, CTH, GY, and HI. A region on chromosome 2B (629.4–781.3 Mbp) harbored MTAs that control CHLD and CTH, overlapped with the GY region spanning 629.4–705.2 Mbp. We found another region on chromosome 3A at 395.8 Mbp that contained MTA for CTH close to the GY MTA at 473.7 Mbp (Figures 4, 6).

We detected two stable markers on chromosomes 4A (rs2252536 at 572.5 Mbp) and 4B (rs2278767 at 30.6 Mbp) for PHT at TOT and DON, respectively. These MTAs explained 11.9–19.3% of the phenotypic variation. An MTA on chromosome 4B (rs1278393 at 32.8 Mbp) had a pleiotropic effect for HI at TOT and CHLD at DON that explained 13.7 and 13.2% of the phenotypic variation, respectively (Table 2).

**Table 2:** Stable and pleiotropic marker-trait associations in the four environments; Tottori (TOT), Dongola (DON), Wad Medani first sowing date (MED/SD1) and Wad Medani second sowing date (MESD/SD2) used to evaluate the multiple derivative lines (MDLs) during the 2019–20 growing season.

Marker	Chromosome	Position (Mbp)	FDR threshold	TOT	DON	MED/SD1	MED/SD2	Allelic effect (%)
rs1062872	1A	522.682366	0.05			TKW	CHLM	15.4–20.0
rs7336178	1A	522.966035	0.05				CHLM, DH	11.8–28.7
rs4406564	1B	629.363030	0.20, 0.05			TKW, DH, DM	DH, DM	12.4–20.3
rs1071015	2A	62.009636	0.05		CHLD	DH	DH, DM	13.3–22.5
rs982956	2A	36.038265	0.05		CHLD	DH, DM	DH, DM	15.5–8.1
rs2252351	2A	35.846102	0.05		CHLD	DH, DM	DH, DM	15.5–18.0
rs1277633	2A	32.676657	0.05		CHLD	DM	DH, DM	15.8–18.8
rs5970682	2A	32.888573	0.20, 0.05		CHLD, DM	DM	DH, DM	12.6–18.6
rs5412116	2B	705.194705	0.05		GY	HTE1		16.0–18.3
rs1151045	2B	724.923696	0.05		CHLD	DM	DH, DM	12.5–21.9
rs984212	3A	713.346253	0.2		CTH		BIO	9.5–11.5
rs2278767	4B	30.576288	0.1, 0.05	PHT	PHT			15.2–19.3
rs1278393	4B	32.888570	0.05	HI	CHLD			13.7–13.2
rs2252536	4A	572.496000	0.05	PHT	PHT			12.9–11.9
rs1018411	6B	160.902903	0.05			GFD	GFD	12.8–15.0
rs1111512	7B	471.484999	0.05, 0.20		CHLD	DM	DH, DM	12.4–21.8
rs1255650	7B	74.684763	0.1, 0.05		CHLD	DM	DH, DM	14.1–22.5

*BIO, biomass; CHLD, chlorophyll degradation; CHLM, chlorophyll at maturity; CTH, canopy temperature at heading; DH, days to heading; DM, days to maturity; GFD, grain filling duration; GY, grain yield; PHT, plant height; TKW, thousand kernel weight*.

### MTAs Under Moderate Heat (MED/SD1) and Severe Heat (MED/SD2) Stress

In MED/SD1, out of 102 MTAs, 94 (92.0%) were for TKW, GFD, or DM. The 37 MTAs identified for TKW explained from 11.2 to 17.3% of phenotypic variation, and four of them were collocated between 496.5 and 522.7 Mbp on chromosome 1A (Figure 4; Supplementary Table S10). Nine MTAs were detected for DM on chromosomes 1B, 2A, 2B, and 7B that explained on average 17.1% of the phenotypic variation. A region on chromosome 1B (629.4 Mbp) harbored an MTA (SNP rs4406564) that affected DH and DM, explaining 12.4 and 20.3% of the phenotypic variation, respectively. Similarly, two of these MTAs collocated on chromosome 2A (35.8–36.0 Mbp) associated with DH and DM, explaining 15.5 and 18.1% of the phenotypic variation, respectively (Figure 4; Supplementary Table S10).

In MED/SD2, we detected 51 MTAs for nine traits, and 38 (74.5%) of them were for BIO, CHLM, DH, or DM (Figure 4; Supplementary Table S10). The eight MTAs for CHLM were located on chromosomes 1A, 2A, 5B, and 6A. Six of these MTAs were collocated with each other: two MTAs on chromosome 1A (522.6–522.9 Mbp) and four on chromosome 2A (754.6–757.1 Mbp). These MTAs explained, on average, 24.4 and 23.3% of the phenotypic variation, respectively (Figure 4; Supplementary Table S10). However, strong MTAs for CHLM were identified on chromosomes 1A (522.9 Mbp) and 5B (528.8 Mbp) that explained 28.8 and 26.8% of the phenotypic variation, respectively (Supplementary Table S10). Furthermore, the MTA for CHLM on chromosome 1A was also associated with DH (Table 2). Among six MTAs for BIO, four were collocated on chromosome 3A (708.3–713.4 Mbp); these explained 11.5–15.4% of the phenotypic variation (Figure 4; Supplementary Table S10). Five MTAs (SNPs rs1071015, rs982956, rs2252351, rs5970682, rs1277633) collocated on chromosome 2A (32.7–62.0 Mbp) controlled DH and DM and explained on average 17.1 and 17.4% of the variation, respectively (Figure 4; Supplementary Table S10). Two MTAs were identified on chromosome 6B, one each for SN (24.6 Mbp) and GFD (160.9 Mbp), explaining 15.1 and 15.0% of the variation, respectively (Figure 4; Supplementary Table S10). Three MTAs for GY were identified on chromosomes 2A (35.6 Mbp), 3A (638.4 Mbp), and 3B (795.3 Mbp); these explained 12.1–15.8% of the phenotypic variation (Figures 4, 5).

A common region on chromosome 2A (32.6–119.8 Mbp) had significant MTAs for DH, DM, HI, and GY. A region on chromosome 3A (590.4–713.3 Mbp) contained MTAs that controlled BIO, DM, GY, and TKW; these MTAs explained 9.0–15.8% of the variation. We identified a region on chromosome 3B (744.0–795.2 Mbp) common to BIO and GY, explaining 12.7 and 14.0% of the allelic variation, respectively (Figures 4, 6).

Several MTAs associated with two or more traits between MED/SD1 and MED/SD2 were identified, indicating a high degree of pleiotropic effects (Table 2). A locus on chromosome 1A (522.6 Mbp) controlled TKW in MED/SD1 and CHLM in MED/SD2. An MTA on chromosome 1B (SNP rs4406564 at 629.4 Mbp) had multiple pleiotropic effects on TKW, DH, and DM in MED/SD1, and on DH and DM in MED/SD2; it explained 12.4–20.3% of the variation in these traits (Table 2).

MTAs associated with multiple traits across environments were also identified in this study (Table 2). A common region on chromosome 2A (33.0–62.0 Mbp) affected CHLD, DH, and DM in DON, MED/SD1, and MED/SD2, respectively, and explained 12.6–22.5% of the phenotypic variation. These traits also showed pleiotropic associations on chromosomes 2B (1 MTA) and 7B (2 MTAs) in DON, MED/SD1, and MED/SD2 with phenotypic variation ranging from 12.5 to 22.5%. We identified another locus on chromosome 3A (713.3 Mbp) that had a pleiotropic effect on CTH in DON and BIO in MED/SD2, explaining 9.5 and 11.5% of the variation, respectively (Table 2). The region on chromosome 1A (358.9–522.6 Mbp) contained MTAs controlling TKW in DON, MED/SD1, and MED/SD2 with phenotypic variation ranging from 11.2 to 16.3%, whereas the region on 6B (81.3–146.7 Mbp) harbored MTAs for TKW in DON and MED/SD1, explaining 17.9 and 15.6% of the allelic variation, respectively (Figure 4; Supplementary Table S10).

### MTAs for Heat Tolerance Efficiency

We calculated HTE based on the GY values under optimum, moderate heat stress, and severe heat stress conditions (Figure 3). For the first HTE (HTE1; calculated from GY of DON and MED/SD1), three MTAs were identified, one located on each of chromosomes 2A (695.9 Mbp), 2B (705.1 Mbp), and 5A (622.3 Mbp); these explained 11.2–20.0% of the phenotypic variation (Figure 4; Supplementary Table S10). For the second HTE (HTE2; calculated from GY of MED/SD1 and MED/SD2), one MTA was detected on chromosome 3A (16.9 Mbp), by using DH as a covariate; it explained 13.3% of the variation (Figure 4; Supplementary Table S10). The locus on chromosome 2B (705.1 Mbp) had a pleiotropic effect on GY in DON and HTE1; it explained 16.0 and 18.3% of the phenotypic variation, respectively (Table 2).

### Effects of Wild Emmer Wheat Alleles in Different Environments

**Figure 7:**
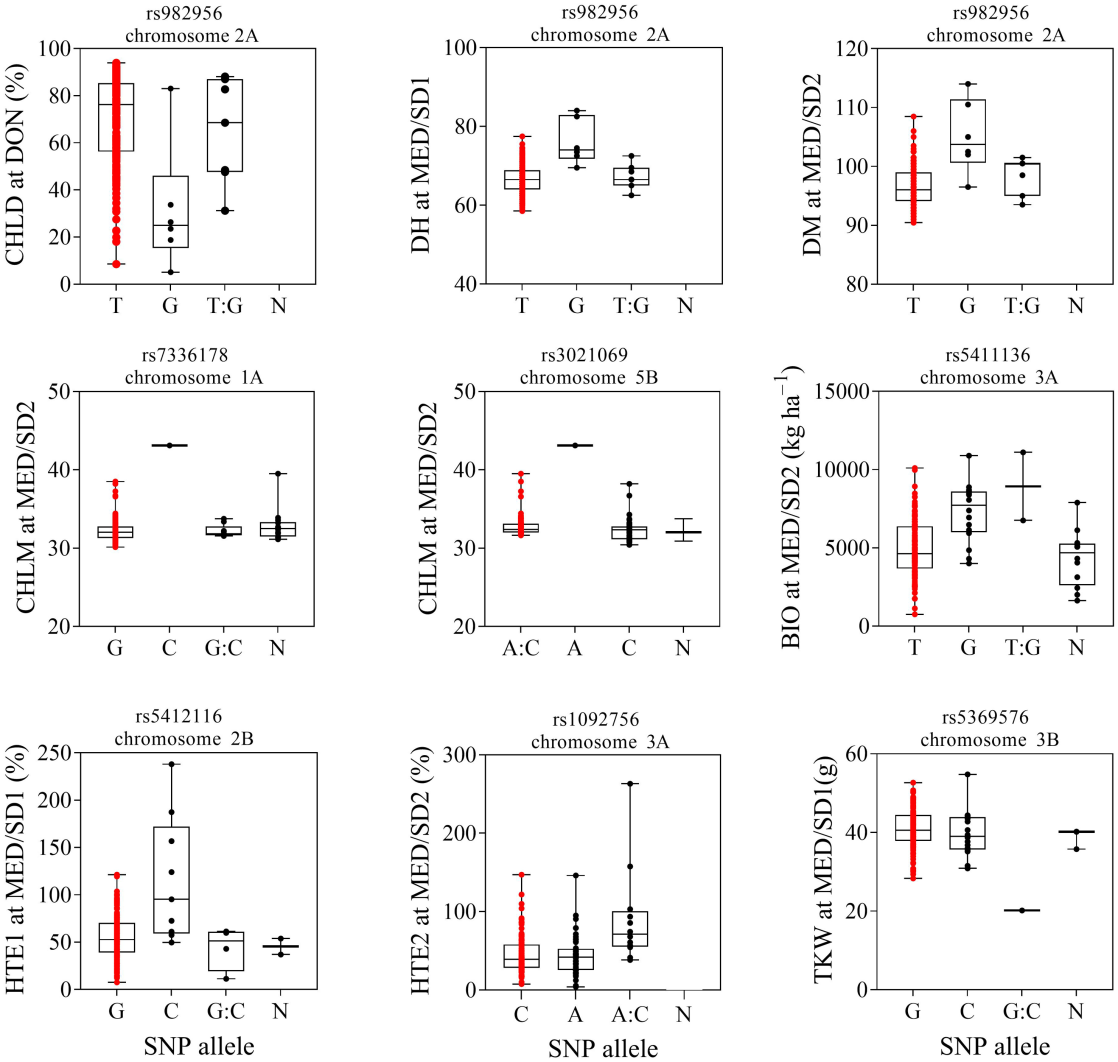
Effect of selected marker-trait associations for different traits evaluated at Dongola (DON), Wad Medani first sowing date (MED/SD1) or Wad Medani second sowing date (MED/SD2). BIO, biomass; CHLD, chlorophyll degradation; CHLM, chlorophyll at maturity; DH, days to heading; DM, days to maturity; HTE1, heat tolerance efficiency evaluated in MED/SD1; HTE2, heat tolerance efficiency evaluated in MED/SD2; TKW, thousand kernel weight. Boxes show median and interquartile range and whiskers show the range. Significant marker and chromosome identified for each trait are displayed on the top box. Red dots indicate genotypes with ‘Miki 3’ allele. A, adenine; C, cytosine; T, thymine; G, guanine; N, unknown.

Strong and pleotropic MTAs were selected to investigate their haplotype diversity across environments (Figure 7). The MTA rs982956 on chromosome 2A had favorable alleles from the wild emmer wheat genome that reduced CHLD in DON, increased DH in MED/SD1, and increased DM in MED/SD2 (Figure 7; Table 2). Under the severe heat stress condition (MED/SD2), we identified two MTAs that had strong positive wild emmer wheat alleles on chromosomes 1A and 5B that increased CHLM: these MTAs explained 28.8 and 26.8% of the phenotypic variation, respectively (Figure 7). Another MTA on chromosome 3A showed a positive SNP allele from the wild emmer wheat genome: it increased BIO and explained 15.4% of the phenotypic variation. We found two MTAs, one each on chromosomes 2B (705.1 Mbp) and 3A (16.9 Mbp) that had positive SNP alleles from wild emmer wheat, increasing HTE1 and HTE2, respectively, compared with the recurrent parent “Miki 3” allele (Figure 7). An MTA located on chromosome 3B had a positive SNP allele from the wild emmer wheat genome that increased TKW in MED/SD1 (Figure 7). To examine the presence of the favorable wild emmer wheat alleles in the elite durum wheat germplasm, we genotyped 43 elite Sudanese durum wheat genotypes and investigated the frequency of the favorable wild emmer wheat alleles in these elite lines (Table 3). Three alleles associated with BIO, CHLM, and TKW were absent from the elite durum lines and the frequency of the other alleles ranged from 2 to 9%. Since the wild emmer wheat has two different lineages, we sought to identify which lineages confer favorable alleles to the MDLs (Table 3). We found that the MDLs carrying a favorable wild emmer wheat allele for CHLM belong to the western lineage, whereas those carrying a favorable allele for BIO belong to the eastern lineage. In contrast, both western and eastern lineages contribute favorable wild emmer wheat alleles for HTE1, HTE2, TKW, CHLD, DH, and DM (Table 3). In this study, we evaluated 43 different phenotypic traits (traits × environments). Out of these 43 traits, only eight (19%) had the positive alleles from the WEW whereas the remaining 35 had their positive alleles from the recurrent parent “Miki 3.” This ratio (19%) is very close to the theoretical ratio of 25% expected from the one backcross event involved in the development of the MDL population (Balla et al., 2022).

**Table 3:** Investigation of wild emmer wheat (WEW) alleles on Sudanese cultivars for some traits showed positive SNP alleles from WEW.

Marker	Chromosome	Trait	Target allele from WEW	Number of Sudanese cultivars that share WEW allele	Number of Sudanese cultivars that share ‘Miki 3’ allele	Number of MDLs belonging to each WEW lineage	
rs7336178	1A	CHLM	C	0	37 (G), 1 (S), 5 (N)	1 Western	
rs5411136	3A	BIO	G	0	43 (T)		16 Eastern
rs5369576	3B	TKW	C	0	41 (G), 2 (N)	5 Western	10 Eastern
rs1092756	3A	HTE2	A:C	1 (A:C)	36 (C), 6 (A)	4 Western	7 Eastern
rs982956	2A	CHLD	G	2 (G)	41 (T)	6 Western	2 Eastern
rs982956	2A	DH	G	2 (G)	41 (T)	4 Western	2 Eastern
rs982956	2A	DM	G	2 (G)	41 (T)	4 Western	2 Eastern
rs5412116	2B	HTE1	C	4 (C)	39 (G)	5 Western	5 Eastern
rs3021069	5B	CHLM	A	1 (A)	32 (M), 10 (C)	1 Western	

*BIO, biomass; CHLD, chlorophyll degradation; DH, days to heading; DM, days to maturity; HTE1, heat tolerance efficiency evaluated in MED/SD1; HTE2, heat tolerance efficiency evaluated in MED/SD2; TKW, thousand kernel weight. A, adenine; C, cytosine; T, thymine; G, guanine; N, unknown*.

### Effect of Allele Combination on GY Under Severe Heat Stress

**Figure 8:**
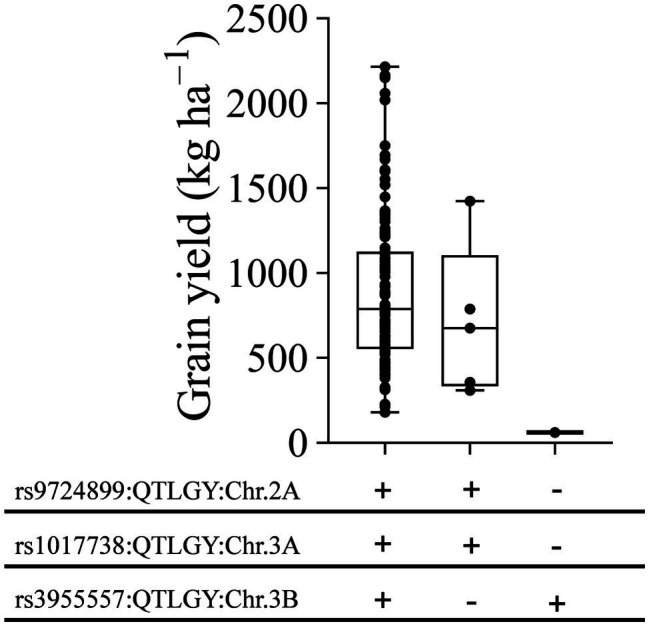
Effect of different allele combinations of significant marker-trait associations on grain yield performance at Wad Medani under late sowing date. The MDLs were divided into three classes based on haplotype diversity analysis for three significant MTAs. Black circles indicate average grain yield by each genotype. “+” marks indicate positive alleles, “–” marks indicate negative alleles. rs9724899, rs1017738, and rs3955557 denote significant MTAs for grain yield on chromosomes 2A, 3A, and 3B, respectively.

Regions on chromosomes 2A, 3A, and 3B regulate multiple traits under severe heat stress, such as DH, DM, GY, BIO, HI, and TKW. We investigated the haplotype diversity at these loci for GY under severe heat stress. Three haplotype classes with different allelic combinations were identified (Figure 8). Combining the positive alleles of these loci showed a wide range of GY from 180 to 2,215 kg ha^−1^. Genotypes with haplotype classes containing two positive SNP alleles on chromosomes 2A and 3A and one negative allele on chromosome 3B had GY ranging from 309 to 1,423 kg ha^−1^. The third class, containing one genotype with two negative alleles on chromosomes 2A and 3A and one positive allele on chromosome 3B, had GY of 60 kg ha^−1^ (Figure 8).

### Candidate Genes Analysis

We searched for the candidate genes of the most important markers identified in the different environments (Supplementary Table S11). Most of the identified genes were related to abiotic stress tolerance, especially heat stress tolerance in bread wheat. A candidate gene *TraesCS2B02G521800* in the region of the pleiotropic marker rs5412116 associated with HTE1 in MED/SD1 and GY in DON on chromosome 2B encodes serine/threonine-protein kinase. It regulates hyperosmotic stress responses and ABA signaling (Mao et al., 2010). A candidate gene *TraesCS3A03G0073100* on chromosome 3A for HTE2 was characterized as gene related to zinc finger family protein and regulates heat stress tolerance in bread wheat (Agarwal and Khurana, 2018). Strong significant MTAs in this study were identified for CHLM on chromosomes 1A and 5B with a phenotypic variation of 28.8 and 26.8%, respectively (Figure 4; Supplementary Table S10). The deep search around sequences of these MTAs for candidate genes showed 136 genes (Supplementary Table S11). Among them, the genes *TraesCS1A02G341400* on chromosome 1A and *TraesCS5B02G351600* and *TraesCS5B02G350900* on chromosome 5B encode for proteins involved in regulating stay-green traits (Tyagi et al., 2017; Zhang et al., 2020; Supplementary Table S11). The candidate genes associated with other yield traits such as BIO, TKW, SN, and HI were also identified (Supplementary Table S11).

## Discussion

### MDL Responses to Heat Stress and Wild Emmer Wheat Contribution to Heat Tolerance

The mechanism of heat stress tolerance is not well studied in durum wheat. We evaluated MDLs over four environments to identify QTLs linked to heat stress tolerance. In the present study, the late sowing environment reduced GY by 82, 77, and 57% compared with TOT, DON, and MED/SD1 and revealed a consistent decrease with the increase in temperature from TOT to DON and MED/SD1 (Figure 1); thus, selecting these environments for evaluation was appropriate as they represent different levels of heat stress. Although heat stress resulted in a severe reduction in GY, some MDL lines were superior and produced higher GY than their recurrent parent “Miki 3” under moderate and severe heat stress (Figure 2), indicating that useful genetic variation was introduced from the wild emmer wheat. The MDLs exhibited highly significant differences for most of the studied traits in all environments as well as G × E interaction, indicating the presence of genetic variability in this panel. The MDLs would respond positively to selection because all traits except CHLM had moderate-to-high heritability, indicating firm genetic control for these traits. Similar results for most traits under optimum and heat stress conditions were reported in bread and durum wheat (Elbashir et al., 2017; Sukumaran et al., 2018; Aberkane et al., 2021; Itam et al., 2021b; Ullah et al., 2021). Low heritability estimates observed for CHLM may be due to a relatively higher error variance in measurements at different environments (Ogbonnaya et al., 2017). Although heat stress drastically reduced most of the studied traits, it significantly increased CHLM compared to the favorable conditions at DON (Supplementary Figure S1). These results indicate that plants in this population maintain high greenness during the maturity stages in response to heat stress.

GY was positively correlated with BIO and SN in all environments, suggesting that selection based on BIO or SN will be adequate to improve GY under optimum or heat stress conditions. Similar results have been reported under similar field conditions except for TOT (Elbashir et al., 2017; Aberkane et al., 2021; Itam et al., 2021b). GY correlated with HI and TKW only under Sudanese environments indicating their importance in yield determination under heat stress. We identified a significant negative correlation between GY and CTH. As a similar result was previously reported under field conditions (Elbashir et al., 2017; Itam et al., 2021b), CTH would be a practical selection criterion to improve GY in dry, hot environments such as Sudan.

In the current study, we used HTE (an indicator of yield loss under heat stress) to identify heat-tolerant genotypes among the MDLs (Figure 3). GY is very important for breeders; therefore, we regressed the HTE to GY to be able to select the tolerant genotypes with good yield performance. For both HTE1 in MED/SD1 and HTE2 in MED/SD2, 5 and 25% of the MDLs, respectively, showed higher HTE than their recurrent parent “Miki 3.” However, except for four genotypes (MDL36, MDL48, MDL87, and MDL117), most of the genotypes with higher HTE had a lower GY than “Miki 3” (Figure 3). Moreover, HTE showed a positive correlation with GY, BIO, and HI, in both moderate and severe heat stress environments and with CHLM only under severe heat stress. Therefore, we attribute the high ratio of tolerant genotypes in MED/SD2 (25%) to the positive correlation between HTE2 and CHLM (Supplementary Table S5). This finding confirms the importance of the stay-green trait as an essential heat tolerance mechanism in wheat (Kamal et al., 2019).

The four high-yielding heat-tolerant MDL genotypes identified are promising and can be integrated into breeding programs to improve heat stress tolerance in durum and bread wheat. The other heat-tolerant genotypes with low yield potential could also be used as a good source of tolerance.

### GWAS and Dissection of the Heat-Associated MTAs

Multi-environment GWA analysis is a practical approach to elucidate the genetic basis of complex traits such as GY and stress tolerance. However, this approach is influenced by the effects of phenology genes on other agronomic traits (Sukumaran et al., 2018). In this study, the identified MTAs for all traits were independent of the effects of plant phenology genes except HTE2. Our panel was pre-selected based on heading under Sudanese conditions (short hot dry growing season); it had a relatively homogenous heading time (Supplementary Figure S1), making it ideal to screen for heat stress tolerance. Several MTAs were identified in all environments, with phenotypic variation ranging from 7.3 to 28.8%. A and B genomes had a similar number of MTAs, consistent with recent findings (Ullah et al., 2021). In this study, the highest number of MTAs was observed on chromosomes 2A and 2B, consistent with the previous findings (Sukumaran et al., 2018) in durum wheat materials. To highlight the usefulness of the MTAs identified for some traits in this study, we compared our findings with the previous GWAS studies in bread wheat (Kumar et al., 2020; Itam et al., 2021b; Ullah et al., 2021) and durum wheat (Sukumaran et al., 2018; El Hassouni et al., 2019; Supplementary Table S12). The MTA position on chromosome 3A (473.7–638.4 Mbp) in DON and MED/SD2 for GY indicates the stability of these regions and its importance under favorable and heat stress conditions; for these reasons, this region could be a target for marker-assisted selection. The other MTAs for GY were located in the distal parts of chromosome 2A (35.6 Mbp in DON and 749.7 Mbp in MED/SD2); thus, the environment affected the position of these QTLs on this chromosome. Similar results were reported for the same chromosomes in bread wheat under heat stress (Kumar et al., 2020; Ullah et al., 2021), and this is the first such report in durum wheat on this chromosomes for the optimum and heat stress environments.

The MTA for GY on chromosome 3B (795.3 Mbp) was found only under the severe heat stress conditions, indicating that this region may provide opportunities to improve GY under heat stress conditions and should be further investigated to validate its suitability for breeding. A QTL affecting GY on the same chromosome was previously reported under late sowing conditions (Bennett et al., 2012; Kumar et al., 2020).

Under severe heat stress, we found GY regions that harbored MTAs clustered with other important yield-related traits (Figure 6). For instance, a locus on chromosome 2A (35.6–119.8 Mbp) controlled GY and HI; a locus on chromosome 3A (631.6–713.4 Mbp) controlled TKW, GY, and BIO; and a locus on chromosome 3B (774.1–795.3 Mbp) controlled BIO and GY. In this study, GY under severe heat stress was positively correlated with BIO, HI, and TKW (Supplementary Table S5). These results indicate that associated traits are likely mapped to similar locations (Liu et al., 2019a) and suggest that BIO, HI, and TKW are effective selection criteria for GY improvement in a heat stress environment. Under the same severe heat stress, we found an independent MTA on chromosome 6B (24.6 Mbp) that controls SN. A similar result was obtained by El Hassouni et al. (2019), who found a QTL associated with grain number per spike under heat stress on the same chromosome in durum wheat inbred lines.

There was a high degree of similarity between the MTAs for DH and DM under heat stress on chromosomes 1B, 2A, 2B, and 7B (Supplementary Table S12). A similar observation was reported by Ullah et al. (2021), who concluded that, under heat stress, the effect of phenology genes is prominent as significant MTAs mapped on chromosomes 2A and 2B, corresponding to the photoperiod genes *Ppd-A1* and *Ppd-B1*. The locations of MTAs on chromosomes 1B and 7B for DH are similar to those reported previously under optimum, heat, and combined heat and drought stresses in bread wheat (Tahmasebi et al., 2016; Schmidt et al., 2020; Ullah et al., 2021), and under optimum conditions for durum wheat materials (Sukumaran et al., 2018).

A strong association among traits was observed within and across environments. This indicates a high level of pleiotropic effects and stability of the QTLs identified across environments in this study, as found by Ullah et al. (2021) in emmer-derived materials. We identified an MTA on chromosome 1A (522.6 Mbp) that controlled TKW in MED/SD1 and CHLM in MED/SD2; this finding suggests that high chlorophyll content at maturity would result in high TKW and greater yield under heat stress. Although we found no positive association of CHLM with other yield-related traits (Supplementary Tables S2−S5), we found a positive trend between CHLM and HTE2 (Supplementary Table S5); thus, the presence of stay-green in these materials led to improved heat stress tolerance. QTLs for CHLM on chromosomes 1A and 5B and HTE2 on 3A explained a great phenotypic variation in this study, and a combination between them will likely increase the degree of tolerance and ultimately improve GY and its component traits. Furthermore, the favored alleles for these QTLs were derived from the wild emmer wheat genome and might not be well represented in elite durum wheat germplasm (Table 3; Figure 7). Therefore, these QTLs for CHLM and HTE2 should be carefully validated in recombinant mapping populations to understand their combined effects on GY and its related traits. It is worth noting that the MTAs for HTE2 on chromosome 3A and CHLM on chromosome 5B were linked to important genes (Supplementary Table S11). For instance, the gene *TraesCS3A03G0073100* for HTE2 on chromosome 3A is annotated as a zinc finger family protein (Supplementary Table S11). This family includes C4HC3-type zinc finger *TaZnF* from bread wheat, its overexpression was reported to increase tolerance to heat, oxidative, and cold stress (Agarwal and Khurana, 2018). Similarly, the gene *TraesCS5B02G351600* identified for CHLM on chromosome 5B encodes superoxide dismutase (Supplementary Table S11). The superoxide dismutase gene from bread wheat *TaSOD* stimulates antioxidant enzymes such as catalase, peroxidase, and ascorbate peroxidase, which are involved in various defenses against reactive oxygen species activities resulted from oxidative stress (Tyagi et al., 2017).

Similarly, markers found on chromosomes 2A, 2B, and 7B, had pleiotropic effects on CHLD, DH, and DM in DON, MED/SD1, and MED/SD2, respectively. This result indicates that low chlorophyll degradation is associated with longer DH and DM and is supported by haplotype diversity analysis of chromosome 2A (SNP rs982956), which shows that genotypes with a positive G allele from the wild emmer wheat genome reduced CHLD and consequently increased DH and DM (Figure 7). Interestingly, this wild emmer wheat allele is not abundant in the elite durum germplasm (Table 3).

A marker on chromosome 2B (705.1 Mbp) had a pleiotropic effect on GY and HTE1 in DON and MED/SD1, respectively. Chromosome 2B was previously reported for GY and tolerance indices (heat susceptibility index) under optimum and heat stress conditions in bread wheat (Qaseem et al., 2019). Although MTAs associated with heat stress index in durum wheat were reported on the same chromosome (Sukumaran et al., 2018), this is the first report of an MTA with pleiotropic effects on GY and stress tolerance index in durum wheat. Interestingly, the candidate gene *TraesCS2B02G521800* associated with this marker encodes for serine/threonine-protein kinase, and regulates hyperosmotic stress response (Supplementary Table S11). Mao et al. (2010) found that the overexpression of *TaSnRK2.4*, an SNF1-type serine/threonine-protein kinase of bread wheat, delayed seedling establishment, promoted longer primary roots, and increased yield under normal growing conditions. Moreover, the *TaSnRK2.4* overexpression enhanced the tolerance to drought, salt, and freezing stress conditions.

We observed an MTA with pleiotropic effects on CTH and BIO in DON and MED/SD2 on chromosome 3A (713.3 Mbp); genotypes with low CTH tend to produce higher BIO. Itam et al. (2021b) reported an association between canopy temperature and BIO located on chromosomes 3D, 5D, and 7D under drought and combined heat and drought stresses in hexaploid wheat. To the best of our knowledge, this is the first report of this genomic region in durum wheat having pleiotropic effects on canopy temperature and BIO. This region could be a potential target for marker-assisted selection after careful validation because both CTH and BIO are associated with GY.

We identified stable MTAs on chromosomes 4A (572.5 Mbp) and 4B (30.5 Mbp) for PHT in TOT and DON. This result indicates the effect of reduced height alleles (*Rht-1*) are prominent in optimum conditions, as these MTAs mapped on chromosomes 4A and 4B are at the same location as reduced height genes *Rht-A1* and *Rht-B1* (Wilhelm et al., 2013). Similar results on the same chromosomes were reported under optimum, heat and combined heat and drought conditions in bread wheat (Itam et al., 2021b; Ullah et al., 2021), and under optimum and heat stress conditions in durum wheat (Sukumaran et al., 2018). Another stable MTA on chromosome 6B (160.9 Mbp) controlled GFD under moderate heat stress (MED/SD1) and severe heat stress (MED/SD2). Similar results were obtained by Sharma et al. (2016), who identified stable QTLs associated with GFD on chromosomes 1B, 2B, 3B, 5A, and 6B under timely and late sowing conditions. This marker could be exploited for molecular wheat breeding programs targeting GFD under heat stress.

The MTA position on chromosomes 1A (359.9–522.6 Mbp) in DON, MED/SD1, and MED/SD2, and on 6B (81.3–146.7 Mbp) in DON and MED/SD1 for TKW indicates stability of these regions under optimum, moderate, and severe heat stress environments. Since TKW is associated with the GY in DON, MED/SD1, and MED/SD2 (Supplementary Tables S3−S5), these regions could be a target for marker-assisted selection for improving TKW and GY under optimum and heat stress environments. The MTAs mapped on chromosome 6B for TKW are equivalent to the hexaploid wheat grain weight gene *TaGW_2_-6B* location (Qin et al., 2014). Similar results were reported for TKW on chromosomes 1A and 6B under optimum, heat, and combined heat and drought stress in bread wheat (Elhadi et al., 2021b; Ullah et al., 2021), and this is the first time such gene reported in durum wheat on this chromosomes under the optimum and heat stress environments.

Under severe heat stress, three regions on chromosomes 2A, 3A, and 3B regulate multiple traits: DH, DM, BIO, HI, GY, and TKW, indicating that these regions are critical for heat stress tolerance. Investigation of haplotype diversity for GY at these loci revealed that the combination of favorable alleles facilitates high GY. Among the MDLs, the top five high-yielding genotypes were confirmed to carry three favorable alleles. These genotypes can be used for potential direct release or used as parents in crossing schemes to incorporate their favorable alleles to improve GY under heat stress conditions. The positive combination of MTAs on chromosomes 2A (SNP rs9724899) and 3A (SNP rs1017738) efficiently increases GY under severe heat stress (Figure 8). Further analysis to validate these MTAs in recombinant mapping populations would be needed to understand their effects on GY.

Our previous research work described the MDL population development (Balla et al., 2022) and showed that the MDL population captured most of the diversity present in the nine wild emmer wheat parents, with great potential for wheat improvement. This study confirms our previous findings that the MDL platform is an effective and practical way to harness the diversity of wild emmer wheat; in particular, the MDL platform enabled us to identify desirable alleles that are absent in elite durum wheat. Furthermore, we identified some MDL lines with favorable wild emmer wheat alleles, and these lines have been selected to improve the diversity of the A and B genomes of bread wheat. Efforts are currently underway to intercross these MDL lines with selected bread wheat lines to develop a new pentaploid wheat population with traits linked to heat stress tolerance. Crossing tetraploid wheat and hexaploid wheat, together with appropriate strategies for evaluating and selecting desirable lines under heat or combined heat and drought stress conditions, will aid in the genetic improvement of both durum wheat and bread wheat.

## Conclusion

In this study, we conducted GWA analysis for agronomic traits under optimum, moderate heat stress, and severe heat stress conditions with a diverse set of MDLs and identified promising MTAs. Stable loci across environments were identified for important agronomic traits such as grain yield and thousand kernel weight. These loci can be further explored for use in marker-assisted selection and gene discovery. Some of the MTAs identified in this study are specific to heat stress and could be targeted in selection to improve heat stress tolerance. Identification of genotypes with favorable alleles and candidate genes from *T. turgidum* ssp*. dicoccoides* for different traits such as BIO, TKW, and CHM demonstrates that MDLs are an effective strategy to explore the diversity of wild emmer wheat to adapt wheat to heat stress. In particular, some favorable wild emmer wheat alleles may be absent or not abundant in elite durum wheat germplasm bread for heat stress environments. The MDL platform used in this study provides valuable genetic materials, alleles, and MTAs that can be good sources to adapt A and B genomes of durum and bread wheat to heat stress conditions.

## Data Availability Statement

The datasets presented in this study can be found in online repositories. The names of the repository/repositories and accession number(s) can be found in the article/Supplementary Material.

## Author Contributions

HT, IT, and YG designed the study. MB performed the genetic analysis and wrote the manuscript with input from YG and NK. IT, MB, and MA performed experiments in Sudan. MB, YG, and NK performed experiments in Japan. MB and NK performed phenotypic data statistical analysis. YG, NK, and HT revised the manuscript. HT supervised the research. All authors contributed to the article and approved the submitted version.

## Funding

This study was funded by the Science and Technology Research Partnership for Sustainable Development (SATREPS) grant JPMJSA1805 by Japan Science and Technology Agency.

## Conflict of Interest

The authors declare that the research was conducted in the absence of any commercial or financial relationships that could be construed as a potential conflict of interest.

## Publisher’s Note

All claims expressed in this article are solely those of the authors and do not necessarily represent those of their affiliated organizations, or those of the publisher, the editors and the reviewers. Any product that may be evaluated in this article, or claim that may be made by its manufacturer, is not guaranteed or endorsed by the publisher.

## Footnotes

^1^ http://genstat.co.uk

^2^ http://bbi.irri.org

^3^ http://www.diversityarrays.com

^4^ https://urgi.versailles.inra.fr/blast

^5^ https://urgi.versailles.inra.fr/jbrowseiwgsc/gmod_jbrowse/

^6^ https://www.uniprot.org/dataset/identifier

## Abbreviations

MDL: Multiple derivative lines
WEW: Wild emmer wheat
TOT: Tottori
DON: Dongola
MED/SD1: Wad Medani first sowing date
MED/SD2: Wad Medani second sowing date
BIO: Biomass
CHLD: Chlorophyll degradation
CHLH: Chlorophyll at heading
CHLM: Chlorophyll at maturity
CTH: Canopy temperature at heading
DH: Days to heading
DM: Days to maturity
GFD: Grain filling duration
GY: Grain yield
HI: Harvest index
HTE1: Heat tolerance efficiency evaluated in MED/SD1
HTE2: Heat tolerance efficiency evaluated in MED/SD2
PHT: Plant height
SN: Seed number/spike
TKW: Thousand kernel weight.
