# Supplementary figures and images for: Exploiting Wild Emmer Wheat Diversity to Improve Wheat A and B Genomes in Breeding for Heat Stress Adaptation
Source: Front Plant Sci. 2022 Jul 22;13:895742. doi: 10.3389/fpls.2022.895742 (PMC9355596; doi:10.3389/fpls.2022.895742)

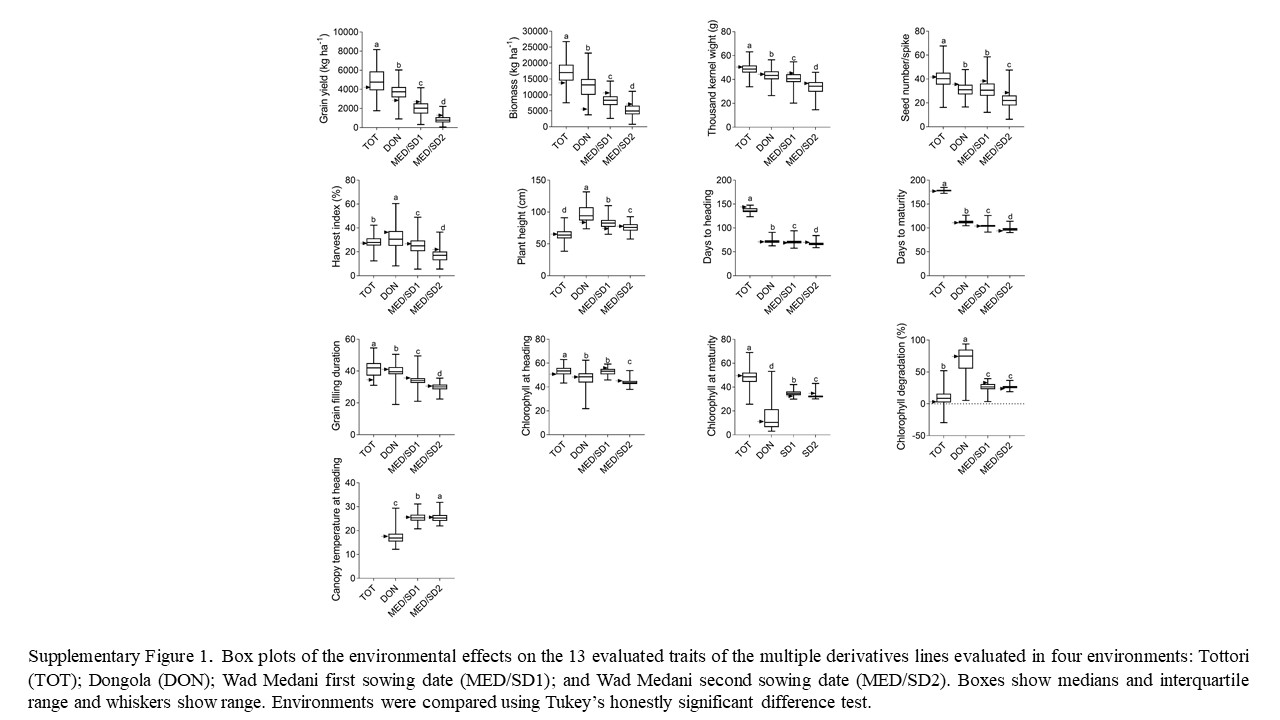

Supplement: Supplementary file 2 [file Image_1.jpg]

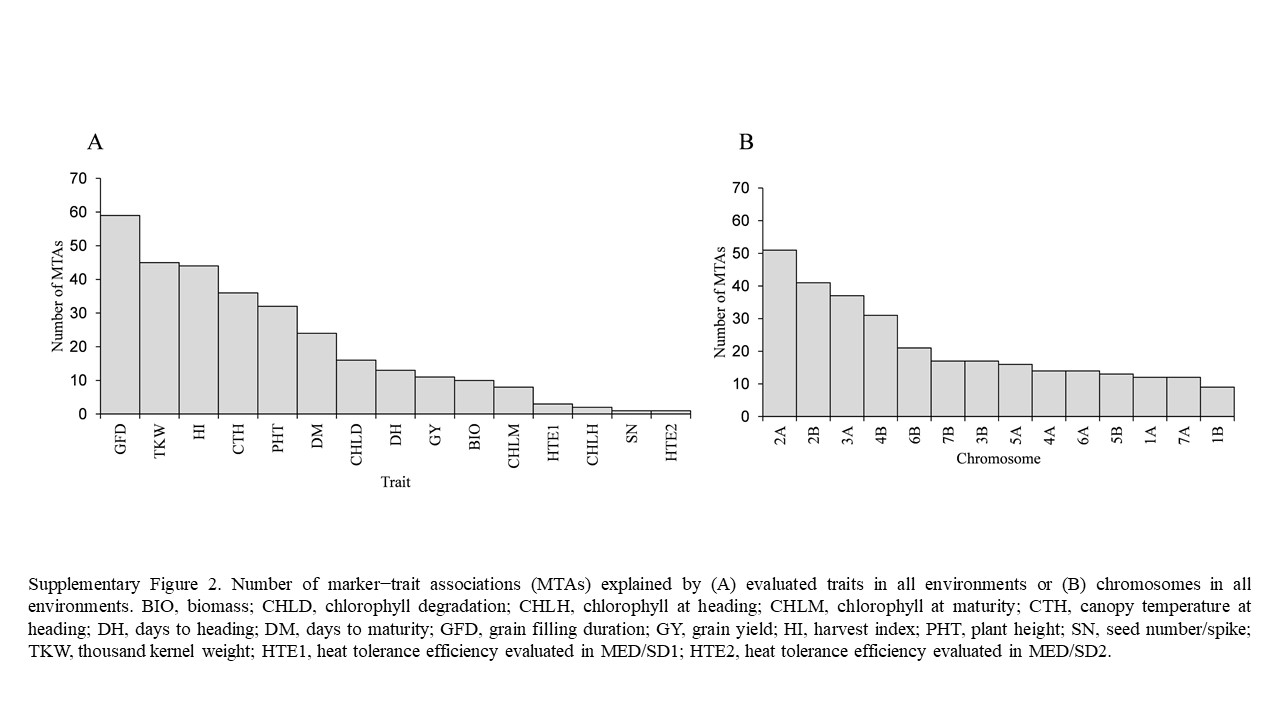

Supplement: Supplementary file 3 [file Image_2.JPEG]
